# Are There Unidentified Factors Involved in the Germination of Nanoprimed Seeds?

**DOI:** 10.3389/fpls.2020.00832

**Published:** 2020-06-10

**Authors:** Umashankar Chandrasekaran, Xiaofeng Luo, Qichao Wang, Kai Shu

**Affiliations:** ^1^Research & Development Institute of Northwestern Polytechnical University in Shenzhen, Shenzhen, China; ^2^School of Ecology and Environment, Northwestern Polytechnical University, Xi'an, China; ^3^Institute of Ecological Agriculture, Sichuan Agricultural University, Chengdu, China

**Keywords:** seed germination, phytohormones, ROS, nanoparticle, seed priming, starch degradation

## Introduction

Nanotechnology-driven smart agriculture has been considered as one of the highly potential approaches in improving crop productivity (Fincheira et al., 2020). Actually, plants serve as a potential pathway for the transportation of nanoparticles (NPs), closely resembling endogenous mineral nutrients. In modern agricultural production system, rapid and uniform seed germination is required for successful seedling establishment and to finally yield achievement (Chen and Arora, 2013). The increasing application of nanoparticles in diverse agricultural sectors has made it a crucial subject of study. Although nanoparticle based studies are fruitful in numerous fields over a decade, such as nanomedicine (Spence et al., 2015; He et al., 2016; Liu et al., 2018), nanoindustrial application (Santos et al., 2015), nanopharmacy (Hsu et al., 2018) and nanopesticide (Sarlak et al., 2014; Kumar et al., 2019), a higher advancement of nanoparticle based studies in releasing seed dormancy and enhancing seed germination as well as seedling development have recently come to the limelight in the form of seed-nanopriming technology.

Seed priming is a process in which partial hydration of a seed is performed using natural/synthetic compounds such as vitamins, PEG or water before sowing (Hussain et al., 2015; Ibrahim, 2016). Nanopriming, a technique based on the combination of seed priming and nanoparticle treatment, has been an useful tool for enhancing seed quality, seedling establishment and crop yields as well as increasing tolerance to environmental stresses, compared to unprimed or other agents primed seeds in tomato, cucumber and pea crops (Mahakham et al., 2017). Nanopriming technology has risen to the limelight only in recent years with reports published in both dicot and monocot seeds (Mahakham et al., 2017; Anand et al., 2019). However, the information provided in the recently published reports are at the preliminary level with depiction of phytohormone crosstalk limited only to abscisic acid (ABA) and gibberellins (GA). These reports have not presented a detailed physiological as well as molecular analysis in relation to the various factors regulating the effect of seed nanopriming on germination. Therefore, the need to understand the detailed molecular mechanisms particularly, the nanoparticle driven other phytohormones (except ABA and GA) biosynthesis and signaling cascades in different primed seed compartments (seed coat, endosperm, and embryo) is of interest given the promotive role of nanopriming towards seed germination. Here, we present some important questions with regard to the unidentified factors in this novel filed.

## Roles of Auxin During Nanoparticle Adhesion in Nanoprimed Seeds

**Figure 1:**
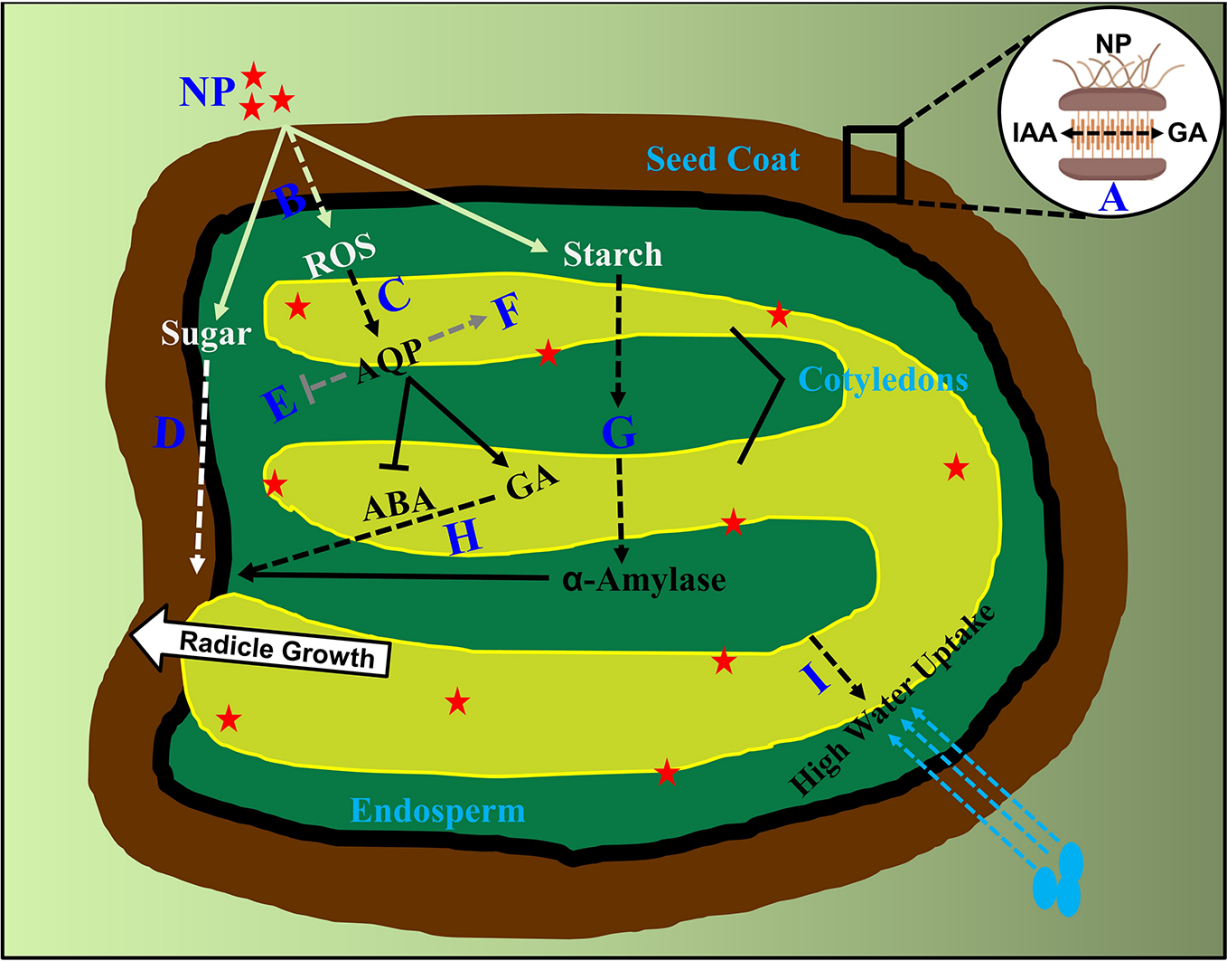
Supposed molecular events occurred in nanoprimed seed compartments. Internalization of nanoparticles from seed coat into nanoprimed seeds. **(A)** Undetermined involvement of GA and IAA in internalization as well as transport from seed coat to endosperm is highlighted by dashed lines. **(B)** Unidentified antioxidant scavengers triggering transduction events in ROS signaling on seed germination **(C)** Unknown downstream regulators of ROS transcription factors in turning on phytohormones is shown.**(D)** Factors involved in sugar signaling responses after nanoparticle adhesion to radicle emergence is indicated. **(E, F)** Phytohormone crosstalk between aquaporin genes and nanoparticles beyond ABA/GA are highlighted which includes the both upregulated and downregulated aquaporin mediated gene transcription. **(G)** Involvement of phytohormones and factors controlling the breakdown of starch granules in to α-amylase after nanoparticle internalization. **(H)** Undetermined downstream regulators of GA signaling pathway in promoting seed germination after priming is denoted. **(I)** Role of phytohormones in high uptake of water in nanoprimed seeds after imbibition is currently unknown and are presented. NP, Nanoparticles; AQP, Aquaporin's; GA, Gibberellic acid; ABA, Abscisic acid. Dashed lines indicate unknown factors involved.

It has been reported that the binding proportion between seeds and priming agent in nanoprimed seeds was found to be high compared to other agent's primed seeds, such as PEG, water and vitamins. (Mahakham et al., 2017; Anand et al., 2019). In relation to this, the seed coat phenolic is endogenously regulated by the hormonal balance of ABA and GA, helping in nutrient passage across seed compartments in *Suaeda salsa* seeds (Xu et al., 2016), whereas on the other side it has also been found that the phytohormone auxin (IAA) produced in endosperm, transport to seed coat in crosstalk with GA by the mediation of AGL62 transcription factor (Figueiredo et al., 2016). From these available evidences, it needs to be first cleared whether IAA have a role in nanoparticle internalization and transport across tissues in primed seeds? If so, how does it interplay with ABA/GA in causing higher percent of nanoparticle adhesion? Further, how do these two hormones or even more regulate the carriers involved in transporting nanoparticles from seed coat to endosperm and then to embryonic tissues, has not been characterized (**Figure 1**). To understand this, an auxin insensitive mutant *msg1*, defective in hypocotyl growth, can be assessed under nanopriming and non-priming treatments (Watahiki and Yamamoto, 1997). The outcome of this experiment would determine the participation of IAA in nanoparticle adhesion and transportation in primed seeds with regard to hypocotyl elongation. A vast study needs to be performed to perceive this action. Further, it will also be of interest, whether the *in vivo* seed mineral content have a role during internalization of various mineral based nanoparticles (for example; iron, silver, copper, zinc and titanium based). To assess this, overall mineral composition and metabolite analysis are required to be assessed in the coming years in nanoprimed seeds.

## Crosstalk Between ROS and Phytohormones in Nanoprimed Seeds

Previous studies have revealed that ABA represses whereas GA enhances seed germination (Shu et al., 2013; Shu et al., 2016a; Shu et al., 2016b; Lorrai et al., 2018; Shu et al., 2018). In addition, a recent study has also highlighted the response of various phytohormones to nanoparticle treatment during plant growth and development (Yang et al., 2017). Intriguingly, seeds recognize nanoparticles as external agents, however the knowledge about this perception process, except for ABA and GA-mediated pathways, are currently unavailable (Mahakham et al., 2017; Anand et al., 2019). In general, nanoparticles internalization on the seed coat induce reactive oxygen species (ROS) accumulation, there by activating several chains of downstream events (Guha et al., 2018). ROS signaling is required for seed dormancy breaking and stimulation of germination probably *via* the activation of GA synthesis and mobilization of storage proteins (Dietz et al., 2016). Wide knowledge about the crosstalk between ROS and phytohormones signaling for dormancy release are currently known, whereas only meagre studies are available on enforcement of ROS in nanoprimed seeds (Oracz and Karpinski, 2016). Notably, the spatial and temporal localization of ROS plays a pivotal role in the cell‐to‐cell communication and the breakage of hydrolytic bonds between polysaccharides in the cell wall of seed endosperm (Oracz and Karpinski, 2016). ROS are efficiently interlinked with the GA and ABA which are associated with seed germination and seed dormancy (Bailly and Kranner, 2011; Bailly, 2019).

From these evidences, we speculate that ROS probably act as a positive signal in the release of seed dormancy, enforced by nanopriming treatment. However, it is to be noted that the knowledge about phytohormones interfering in ROS influx in intercellular trafficking other than ABA/GA are largely unknown (Mahakham et al., 2017). Hence, it remains to be explored in knowing about the downstream protein targets modified by ROS as well the transporters involved in intercellular transportation, enabling stimulus specific cellular responses from the seed coat or the molecular regulators which allow ROS–phytohormone interactions tuning in seed germination including ABA, GA, auxin, and other hormones (**Figure 1**). In association to this, a complete genomic as well as proteomic analysis needs to be performed using ROS signaling mutants to determine the roles of specific ROS related enzymes participating in the crosstalk networks.

## Role of Antioxidant Scavenging System in Promoting Germination in Nanoprimed Seeds

Seeds must be well supported by a scavenging system that tightly regulates ROS concentration and enables them to act as cellular messengers. Previous studies reported the accumulation of ROS, e.g. hydrogen peroxide (H_2_O_2_), hydroxyl radicals (OH) and superoxide radicals (O2) that enhanced the dynamics of seed germination in various crops (Wojtyla et al., 2016). ROS induced upon external stimuli is fine-tuned by the antioxidant system (Mittler et al., 2011). By maintaining ROS homeostasis, the antioxidants system plays an important role in redox regulation by ROS removal and counteracts potential molecular damage in cells (Dietz et al., 2016). This system involves several antioxidant enzymes, such as guaiacol peroxidase (POX), catalases (CATs), and superoxide dismutases (SODs) and enzymes of the ascorbate-glutathione cycle, such as ascorbate peroxidase (APX), dehydroascorbate reductase (DHR), and glutathione reductase (RG), in association with other low-molecular-weight antioxidants like ascorbic acid, glutathione (GSH) and its oxidized form glutathione disulfide (GSSG) (Gupta et al., 2019).

The antioxidant enzymes indirectly determine the role of ROS in promoting germination in nanoprimed seeds (Elmaaroufbouteau et al., 2013; Anand et al., 2019). For example, activity of SOD and CAT was observed to be increased thereby controlling the activity of “H_2_O_2_” radicals in tomato, cucumber and pea nanoprimed seeds (Barbaespin et al., 2012; Bhardwaj et al., 2012; Anand et al., 2019). ROS accumulation in the form of O_2_ and H_2_O_2_ radicals play a positive role in the germination and dormancy release (Mittler et al., 2011). From the available evidences, it is clear that the antioxidant regulation of ROS is limited to SOD and CAT enzymes in diverse nanoprimed seeds, thus requiring a vast exploration in understanding the regulatory mechanism of ROS accumulation by the other antioxidant scavengers. In addition, H_2_O_2_ also regulates the expression of various genes involved in the germination process, through protein carbonylation, activation, and modulation of kinase transduction cascades along with changes in the cellular redox states (Elmaaroufbouteau et al., 2013). In relation to this, we speculate that the participation of antioxidant scavengers in triggering these transduction events needs further elucidation. Besides these antioxidant enzymes, participation of a metal binding protein metallothionein (MT) as an H_2_O_2_ scavenger has also been reported (Zhou et al., 2012; Leszczyszyn et al., 2013; Mierekadamska et al., 2018). Expression of two metellothinein genes *MT1* and *MT4* were found be highly induced in nanoprimed tomato seeds, suggesting their possible involvement in ROS signaling during germination of nanoprimed seeds (Anand et al., 2019). However due to the limited experimental evidences, further studies involving metallothionein related mutants might provide the evidence for elucidating the role of metallothionein in the scavenging ROS signaling in nanoprimed seeds.

## Phytohormones Mediated Starch and Sucrose Metabolism in Nanoprimed Seeds

Nanopriming involves rapid starch degradation, determined in terms of *α*-amylase activity (Mahakham et al., 2017). In line with this, another study found that α-amylase pitched the starch granule surface first, then penetrated into the interior and hydrolyzed the granule from the inside out, implying a higher induction of α-amylase activity in nanoprimed seeds (Man et al., 2013). This induction of biosynthesis of α-amylase is dependent on the activity of GA. Evidently, Mahakham and colleagues, showed the failure in the production of α-amylase under the absence of GA (Mahakham et al., 2017). This study clearly states a signaling crosstalk pathway existing among nanoparticles, a-amylase and GA in nanoprimed seeds. However, the upstream GA signaling factors involved in the starch degradation *via* α-amylase activity are yet to be identified. Intriguingly, a previous study showed an existence of antagonism between GA and cytokinins in regulating α-amylase activity during metal (cadmium) stress (Atici et al., 2005). However, it remains unclear on the involvement of multiple phytohormones in α -amylase enrichment in nanoprimed seeds. In addition to this, it will also be interesting to investigate the crosstalk between phytohormones and sugar signaling responses initiated by nanoparticles after priming (**Figure 1**). To further bisect, involvement of sugar insensitive mutants like *sis4* and *sis5* which are defective in ABA biosynthesis might be helpful in extracting other phytohormone factors involvement in sugar signaling factors enhancing germination process in nanoprimed seeds (Laby et al., 2000). Future studies involving an overall phytohormone profiling will identify crucial hormones involved in α-amylase enrichment apart from GA. Detailed studies concerning this might produce many number of interesting findings in the coming years.

## Unidentified Factors or Mechanisms Underlying Rapid Water Uptake in Nanoprimed Seeds

Nanopriming treatment can improve seed water uptake, as primed seeds exhibit a faster imbibition in comparison with non-primed as well as other primed agent seeds (Mahakham et al., 2017). Water uptake in seeds is influenced by the balance between ABA and GA, and this ABA/GA balance regulates dormancy induction and release, resulting in shifting water potential thresholds for radicle emergence (Rodriguezgacio et al., 2009). Interestingly, enhanced ROS levels also activate aquaporin signaling pathway genes as well as in causing changes at phosphorylation sites in critical aquaporin proteins rendering a high uptake of water (Boursiac et al., 2008). Critical aquaporin family genes like *PIP2*, *NIP1*, *TIP3* and *TIP4* are controlled by ABA during seed germination (Footitt et al., 2019). How do ABA/GA balance regulate aquaporin genes mitigating faster uptake of water driven by nanopriming? Considering the fact that primed seeds exhibit a faster water uptake by the upregulation of transcription of *PIP1* and *PIP2* (Mahakham et al., 2017), it remains to be cleared about the role of ABA on the control of aquaporin genes expression. Critical transcription factors directly regulating the expression of these genes are needed to be identified (**Figure 1**), as some seed specific vacuolar aquaporin's are regulated by ABI3 transcription factor (Mao and Sun, 2015). In support, it has also been previously established that several phytohormones regulate various plant aquaporin's (Kapilan et al., 2018). Hence, a complete genome wide transcriptome analysis in diverse nanoparticle treatments will be helpful in understanding the common regulatory networks responding to nanoparticles. Also, using aquaporin family of mutants like *pip1*, *pip2*, *tip1*, *nip1* and *pip1pip2* under nanopriming treatments, might unravel many more transcription co-factors associated with aquaporin genes expression in primed seeds. It will also be of vital importance to find the phosphorylation dependent PIP and TIP aquaporin intercellular trafficking triggered by nanoparticles in primed seeds causing an increase in water uptake.

## Conclusion and Future Prospects

Overall, the link between nanoparticle adhesion and phytohormone crosstalk in influencing seed germination in a nanoprimed seed is only in the beginner's stage. Having done several studies on phytohormone crosstalk during seed germination and seedling establishment (Shu et al., 2013; Shu et al., 2016a; Shu et al., 2018), we propose that this emerging field has the potential to: (i) identify the crosstalk between auxin and GA in nanoparticle adhesion in seed coat; (ii) identify downstream regulatory proteins of ROS-Phytohormone complex along with critical antioxidant enzymes; (iii) identify antioxidant scavengers involved in transduction of ROS signaling cascades; (iv) identify phytohormone mediated critical factors that can help in starch degradation during seed germination mediated by nanopriming; (v) find factors and phosphorylation events controlling water uptake and transport mediated by phytohormone and ROS signaling in nanoprimed seeds. These investigations will help us to understand more about the regulatory role of nanopriming in tackling seed germination under severe environmental stress conditions.

## Author Contributions

UC and KS designed the opinion. LX and WQ helped in providing the inputs. UC and KS wrote the manuscript.

## Conflict of Interest

The authors declare that the research was conducted in the absence of any commercial or financial relationships that could be construed as a potential conflict of interest.
